# Responsibility of psychiatrists: Need for pragmatic idealism

**DOI:** 10.4103/0019-5545.31551

**Published:** 2006

**Authors:** Nimesh G. Desai

The etymological basis of ‘responsibility’ is to do with the ‘ability to respond’. It may be added that the response be an able, adequate response and tailored to the ‘need’. It is my contention that the increasing acceptance of mental health as a scientific discipline, and the potential it has for alleviation of human suffering, places the professionals of mental health in a situation wherein awareness about the responsibility is inescapable. Internationally, this has been the regular refrain most notably in the writings on ethical aspects of psychiatric practice. Paul Chodoff identified four major responsibilities of psychiatrists, viz. (i) the responsibility of competence or the need to master their task; (ii) the responsibility of ethical behaviour or to police their ranks; (iii) the responsibility of accountability or to be accountable to the public; and (iv) the responsibility of advocacy or to be advocates for the mentally ill persons.^1^ Lazarus concurred with the views of Chodoff and, while saying that ‘it is likely that the twenty-first century psychiatrist will occupy new roles but continue in the clinical treatment of the mentally ill’, suggested the following guidelines to consider (both for psychiatrists and the system in which they work):^2^ (i) psychiatrists have an ethical obligation to rise above any profit motive and serve as patient advocates; (ii) psychiatrists should provide treatments that are potentially beneficial and chosen by informed patients; (iii) beneficial psychiatric treatments should include those empirically demonstrated to provide benefit that is valued by patients, regarded as part of standard of care, and recommended by established practice guidelines; (iv) if allocation decisions are made, the manner in which these decisions are made and the influence of the treatment philosophy on patient care should be disclosed to patients in a clear manner in advance; (v) treatment decisions should, to as great an extent possible, continue to be a joint enterprise between psychiatrist and patient; (vi) psychiatrists serving in administrative roles have the same professional ethics as clinicians. If they choose to follow a different set of ethics, they are ethically obliged to disclose their ethical standards to other physicians and patients; (vii) organized systems of care should foster a spirit of patient advocacy by professionals who are treating patients; (viii) if there is an obligation assumed by a system of care to conserve society's resources, then all involved (patients, professionals, administrators and other stakeholders) should benefit, profit, or be adversely affected by making proportionate gains or sacrifices; (ix) psychiatrists must continue to behave in a manner that will maintain trust in the doctor–patient relationship and not engage in behaviour that will harm the patient; (x) psychiatrists should be actively involved in any social policy debates that affect psychiatric patients; (xi) psychiatrists must advocate for fair treatment of psychiatric patients in all healthcare systems.

In the Indian context, concerns have been expressed by individual professionals and the Indian Psychiatric Society about the basic issues of ethics in psychiatric practice as well as the need to identify responsibilities of psychiatrists. The Indian Psychiatric Society had adopted a code of Ethics for Psychiatrists in 1992, outlining the major principles of (i) responsibility, (ii) competence, (iii) benevolence, (iv) moral standards, (v) patient welfare, and (vi) confidentiality. A number of Presidential Addresses have focused on various aspects of the subject.^3^–^6^

## PROFESSIONAL RESPONSIBILITIES

The duty to be a competent professional is the most fundamental obligation, and yet is important enough to be stated in the context of India and many other developing countries. The need to emphasize it is in the context of the lack of uniform standards for obtaining the requisite qualification and the lack of monitoring of standards. The perception, indeed the myth, that psychiatry or mental health, as disciplines, are not scientific or rigorous adds to the concern for professional competence. It must be said that the perceived and/or alleged ‘safe’ nature of practice of mental health, with no definitive harm or negative consequences, adds to the complacence of the professionals. The soft nature of the skills involved in mental health practice and the variance in the requisite skills further reduces the perceived need for a level of competence to be attained. The scientific and vigorous nature of mental health practice needs to form the bedrock for accepting the need for professional competence, across the board for trainees, trainers and full-time practitioners.

As with the issue of professional competence, the issue of continuing professional advancement, and, at the very least, the maintenance of professional competence is in need of being recognized and emphasized. The facts that mental health is a relatively new field, that it is undergoing rapid advance-ments in theory and practice, as well as that a wide range of disciplines interface with it, make the task of professional advancement fairly daunting. Nonetheless, it needs to be intensified as one of the obligatory responsibilities, albeit as per the chosen career path and the setting of one's work, within the limitations. Technological advancements in the recent past have made this task easier than it was, with easier transport and communications.

The need for mental health professionals, especially in the developing countries, to be able to project and assert appropriate identity is difficult and yet important. One of the commonest issues, often not realized, is of being a ‘professional’, since the community often does not fully comprehend or accept the mental health as a full-time or scientific profession. Most psychiatrists seem to have had, individually and in groups, issues about their identity vis-à-vis other medical specialties. The perceived, and often externally attributed, ‘non-scientific’ or ‘non-medical’ nature of their work often leads psychiatrists to internal doubts and, sometimes, negative attitudes towards their own profession. The alleged ‘non-effectiveness’ of the treatment methods, and sometimes even the totally misplaced sense of therapeutic nihilism, makes psychiatrists and other mental health professionals almost apologetic about their profession. Non-psychiatry mental health professionals are even more in doubt about their professional identity compared to the psychiatrists. Here again, the lack of adequate acceptance of their professional role and identity, coupled with the limited career options, makes it a difficult task for these mental health professions to develop and assert an appropriate identity. It must be said that ignorant and indifferent, if not negative, attitudes of psychiatrists do not make the task any easier. It should be considered a desirable responsibility of all mental health professionals to be able to develop and project the correct professional identity.

## LEGAL RESPONSIBILITIES

As one of the fundamental requirements of civil society, adherence to law is the responsibility of all citizens, more so of professionals about their own work. It is often misunder-stood that the legal responsibilities are limited to actions to protect oneself. It must be said that it is inescapable for a professional to execute the responsibilities of the profession as per the law. The legal responsibility of mental health professionals can be further subdivided into laws with direct influence on mental health issues, human rights issues, and laws with indirect or implicit influence on mental health issues.

The issues of human rights and mental health have assumed a high significance in the past few years and are starting to occupy the central position in the relationship between the mental health service provider and the consumer. It is often not recognized that the governments of various nations are obligated for such protection under the United Nations Charter of Human Rights.^7^ The responsibility for awareness of and working according to contemporary laws and statues, with provisions thereof, is to be executed not only to protect one's interest, but also as a fundamental obligation.

Since the sweep of mental health issues is rather broad, the laws which have indirect or implicit influence on mental health are many. The Persons with Disabilities Act, 1995, the Narcotics and Psychotropic Substances Act, 1987, Dowry Act, 1961, the Hindu Marriage Act, 1955, the Juvenile Justice Act, 2000, and other similar Acts are, in the opinion of many mental health professionals, not central to their functioning, and so not essential to know in detail. Conversely, it can be argued that since these laws have an indirect but significant influence on mental health issues, it should be considered essential to be familiar with them.

## ETHICAL RESPONSIBILITIES

Ethical responsibilities of mental health professionals have become increasingly focused on with the changing social context of mental health service delivery and the recognition for well regulated code of ethics. There are many difficulties and obstacles in appropriate recognition of ethical responsibility of all health professionals, and particularly mental health professionals in developing countries. These relate to the absence of clear ethical guidelines, lack of consistent application, the attitude of professionals involved, and the still limited assertion of the ethical rights by the consumers.

The responsibility of all mental health professionals to practice according to the guidelines prescribed by various professional bodies is obvious. However, there is a need for a clear set of guidelines, and a straightforward monitoring mechanism. There is also a need for advocacy in this aspect by mental health professionals. The need for not only advocating, but also striving for continuous review and upgradation of the standards of ethical practice, is high and requires to be focused upon by at least some mental health professionals.

## SOCIAL RESPONSIBILITIES

The concept of social responsibilities of psychiatrists or all mental health professionals has been relatively less in focus, and often not recognized or understood. The possibility of debate and disagreement about these responsibilities is certainly larger, and the contextual reference for these responsibilities in developing countries is different from that of developed countries. It may be stated briefly but most assertively that the task of working towards mental health awareness, in different forms, must be seen as an important responsibility, especially in the context of developing countries. The reluctance, if not resistance, on part of some of the mental health professionals, specially those in influential positions, is highly undesirable and needs to be worked through by the concerned professionals or by peer pressure and community participation.

Society's expectation and mental health professionals’ own self-imposed burden about their own mental health has to be effectively tackled to achieve a realistic orientation and acceptance. The belief and expectation that mental health professionals should be entirely free of any mental health problems and ‘above it all’, is generated and contributed to by many factors. It may not be possible to discuss all of them here, but the important ones to recognize are: (i) Society's expectations from all health professionals to be free of any ailment, especially the ones they treat; (ii) the mental health professionals’ own misplaced grandiose belief that the ability to understand human behaviour gives them the capacity to overcome any problem with total equanimity; (iii) some legacy from the Freudian period; and above all (iv) the lack of adequate in-depth acceptance of the concept of mental health by mental health professionals. It is good to ask ourselves as to how many mental health professionals would accept the possibility of suffering from psychological problems or mental health problems? Or accept the need for external help? The tendency, of all health professionals, of ‘playing God’ with other's lives also leads to a kind of feeling or cognitive set of being ‘superhuman’. Unfortunately, the extent of damage it does to individuals and families in question, and also to the identity of mental health as a profession, is often not realized. It is accepted that the possibility of occurrence of mental health problems is as much, if not more, as some people believe, as that in the general population. It should certainly be expected that mental health professionals deal with mental health problems in themselves and in their family members more effectively. Indeed, acceptance of the problem and the need for help is the first essential step in being able to do so.

A related important concern is of interpersonal issues among mental health professionals, in formal or informal groups. It may be worthwhile examining the nature and extent of these problems, and more importantly the ways these are tackled. It is quite possible that the expectations are unduly high and/or that colleagues in other disciplines judge psychiatrists too minutely or harshly. At the same time, it needs to be accepted that the expectation that mental health professionals ought to be able to tackle their own interpersonal issues more effectively is difficult to refute. One of the operative processes is since many, if not most, mental health professionals tend to mistakenly believe, knowingly or otherwise, that their ability to understand human behaviour, interpersonal processes and group dynamics, also makes them capable of being able to do so with their own problems. It is obvious that when most people in a group believe and behave so, it becomes extremely difficult to achieve harmony. It is also interesting to notice how mental health professionals use the terms like ‘paranoid’, or ‘neurotic’ or other such terms for each other, directly or indirectly. Here again, granting that such phenomena happen in many professions, there is a strong possibility that psychiatrists and other mental health professionals are actually a lot more ‘judgmental’ of each other than others.

The need to acquire an adequate, if not complete, understanding of oneself and the society for effective execution of many of the obligatory responsibilities is obvious. In the context of Maslow's ‘Hierarchy of needs’, it may well be said that the expectation from mental health professionals to be able to move towards self-actualization, at least to a certain degree, cannot be argued against.^8^ The other model which may be useful is Johari Window.^9^ The possibility of applications of this model of different parts of a person's self in mental health practice are tremendous and almost unexplored, not just in the limited context of its potential in helping mental health professionals to understand themselves. The exploration of the 2nd (Unknown to Self, Known to Others) and the 3rd (Known to Self, Unknown to Others) quadrants of one's own self can be a very useful exercise for anyone, and certainly for a mental health professional.^10^

## EPILOGUE

In addition, openness to understand how society views the profession, as well as the changing trends in society, seems crucial for mental health professionals. It may not be an exaggeration to say that the mental health profession needs to be aware of all aspects of human existence and the way they impact on mental health in one way or another. The restricted view taken by some psychiatrists in limiting the scope of the profession to medical issues is rather unfortunate and harmful.

There are many dilemmas faced by anyone contemplating seriously on the subject of responsibility of mental health professionals. Some of the important ones are: (i) scope of field of mental health: Does it include newer areas such as HIV prevention, tobacco cessation, suicidal behaviour, domestic violence, etc.?; (ii) subspecializations and superspecializa-tions: Are they necessary or avoidable?; (iii) advocacy versus activism: Can one become an activist, while continuing to be a professional?; (iv) consumer movements, and user and cases groups: While certainly welcome, what impact will they have? Are the professionals ready to work with them in partnership?

In view of the possible differences in opinion among the psychiatrists about the concept of responsibility, a detailed analysis has been made of the professional, legal, ethical and social responsibilities.^11^ The responsibilities range from those that can be seen as being absolutely mandatory or obligatory to those that may be considered optional, with some responsibilities being seen as desirable. The range of responsibilities can be seen as three concentric circles of ‘Must Do’ (Obligatory), ‘Should Do’ (Desirable) and ‘Can Do’ (Optional). The full range of responsibilities is better seen in a pragmatic, stratified manner, across the three levels. The three levels are seen as concentric circles with the obligatory ‘Must Do’ as the core centre, moving through desirable (Should Do) to the optional responsibilities (Can Do).^11^ The three levels also need to be seen as sequential, i.e. the second or third level of responsibilities be attempted only after executing the first level responsibility well. The pragmatic, stratified model suggested has to be seen against the possibility of either an all-encompassing approach, wherein the expectations are far too many and unrealistic, and a restrictive and constraining approach, wherein the boundaries are so rigid as to be totally devoid of the social perspective carried by mental health professionals, the social context, and the willingness and sensitivity of professionals. The delineation of various responsibilities in these three concentric circles will, quite possibly, vary across countries and cultures, or even within the same country or culture. The variance in opinion is understandably likely to be larger as one moves from the centre to the periphery, in this model.

There has been valid criticism of psychiatrists having been too ambitious in issues of social harmony or political stability.^5^ These need to be borne in mind, and yet the possibility of contributing to the processes of social change and harmony, through research and advocacy, should not be overlooked. The limitations of mental health professionals in this direction should be definitely accepted and yet the baby should not be thrown out with the bathwater! The need is for balancing idealism with pragmatism of how much is feasible and how much should be attempted. As Flaherty and Astrachan commented, ‘The dangers of overpromising and boundary diffusions are great, but the perils of passivity are even greater’.^12^
